# Synthesis, crystal structure and thermal behavior of tetra­kis­(3-cyano­pyridine *N*-oxide-κ*O*)bis­(thio­cyanato-κ*N*)cobalt(II), which shows strong pseudo­symmetry

**DOI:** 10.1107/S2056989023006862

**Published:** 2023-09-08

**Authors:** Christian Näther, Inke Jess

**Affiliations:** a Institut für Anorganische Chemie, Universität Kiel, Germany; University of Aberdeen, United Kingdom

**Keywords:** synthesis, coordination compound, cobalt thio­cyanate, 3-cyano­pyridine *N*-oxide, crystal structure, pseudosymmetry, thermal properties

## Abstract

The title compound, [Co(SCN)_2_(C_6_H_4_N_2_O)_4_], was prepared by the reaction of cobalt(II)thio­cyanate with 3-cyano­pyridine *N*-oxide in ethanol. In the crystal, the cobalt(II) cations are octa­hedrally coordinated by two terminal N-bonded thio­cyanate anions and four O-bonded 3-cyano­pyridine *N*-oxide coligands, forming discrete complexes that are located on centers of inversion, hence forming *trans*-CoN_2_O_4_ octa­hedra. The structure refinement was performed in the monoclinic space group *P*2_1_/*n*, for which a potential lattice translation and new symmetry elements with a fit of 100% is suggested. The structure can easily be refined in the space group *I*2/*m*, where the complexes have 2/*m* symmetry. However, nearly all of the reflections that violate the centering are observed with significant intensity and the refinement in *P*2_1_/*n* leads to significantly lower *R*(*F*) values (0.027 *versus* 0.033). Moreover, in *I*2/*m* much larger components of the anisotropic displacement parameters are observed and therefore, the crystal structure is presented in the primitive unit cell. IR investigations confirm that the anionic ligands are only terminally bonded and that the cyano group is not involved in the metal coordination. PXRD investigations show that a pure crystalline phase has been obtained and measurements using simultaneously thermogravimetry and differential thermoanalysis reveal that the compound decomposes in an exothermic reaction upon heating, without the formation of a coligand-deficient inter­mediate phase.

## Abstract 2

The crystal structure of the title compound consists of discrete complexes in which the Co^II^ cations are octa­hedrally coordinated and show strong pseudosymmetry.

## 1. Chemical context

The synthesis of new coordination compounds is still an important field in chemistry. In most cases, such compounds are prepared in solution but there are alternatives, where they are prepared in the solid state using, for example, mol­ecular milling (Stolar *et al.*, 2017 ▸; Darwish *et al.*, 2019 ▸), grinding (Adams *et al.*, 2007 ▸) or molten-flux synthesis (Höller *et al.*, 2010 ▸; Schönfeld *et al.*, 2012 ▸). In our own investigations, we frequently use thermal ligand removal of suitable precursor compounds for the solid-state synthesis of new coordination compounds that mostly consist of discrete complexes, in which the anionic ligands are only terminally bonded. Upon heating, these precursors frequently lose their neutral coligands in a stepwise fashion, forming inter­mediate compounds with condensed networks in which the metal cations are linked by the anionic ligands into one-, two- or three-dimensional networks. In the beginning, our inter­est focused on transition-metal–halide coordination compounds (Näther *et al.*, 2001 ▸; Näther & Jess, 2004 ▸), but in recent years we have used this approach for the synthesis of transition-metal thio- and seleno­cyanates because these anionic ligands mediate reasonable magnetic exchange, which allows the preparation of compounds that show versatile magnetic behavior (Palion-Gazda *et al.*, 2015 ▸; Mekuimemba, *et al.*, 2018 ▸). In this context, of special inter­est are compounds based on Co^II^ in which the cations are linked by pairs of thio- or seleno­cyanate anions into chains, because they can show three-dimensional but especially one-dimensional magnetic ordering (Werner *et al.*, 2014 ▸; Rams *et al.*, 2020 ▸). The major advantage of our approach is the fact that the new compounds are obtained in qu­anti­tative yield and that frequently metastable polymorphs or isomers can be prepared that often are not available from solution (Werner *et al.*, 2015 ▸). 
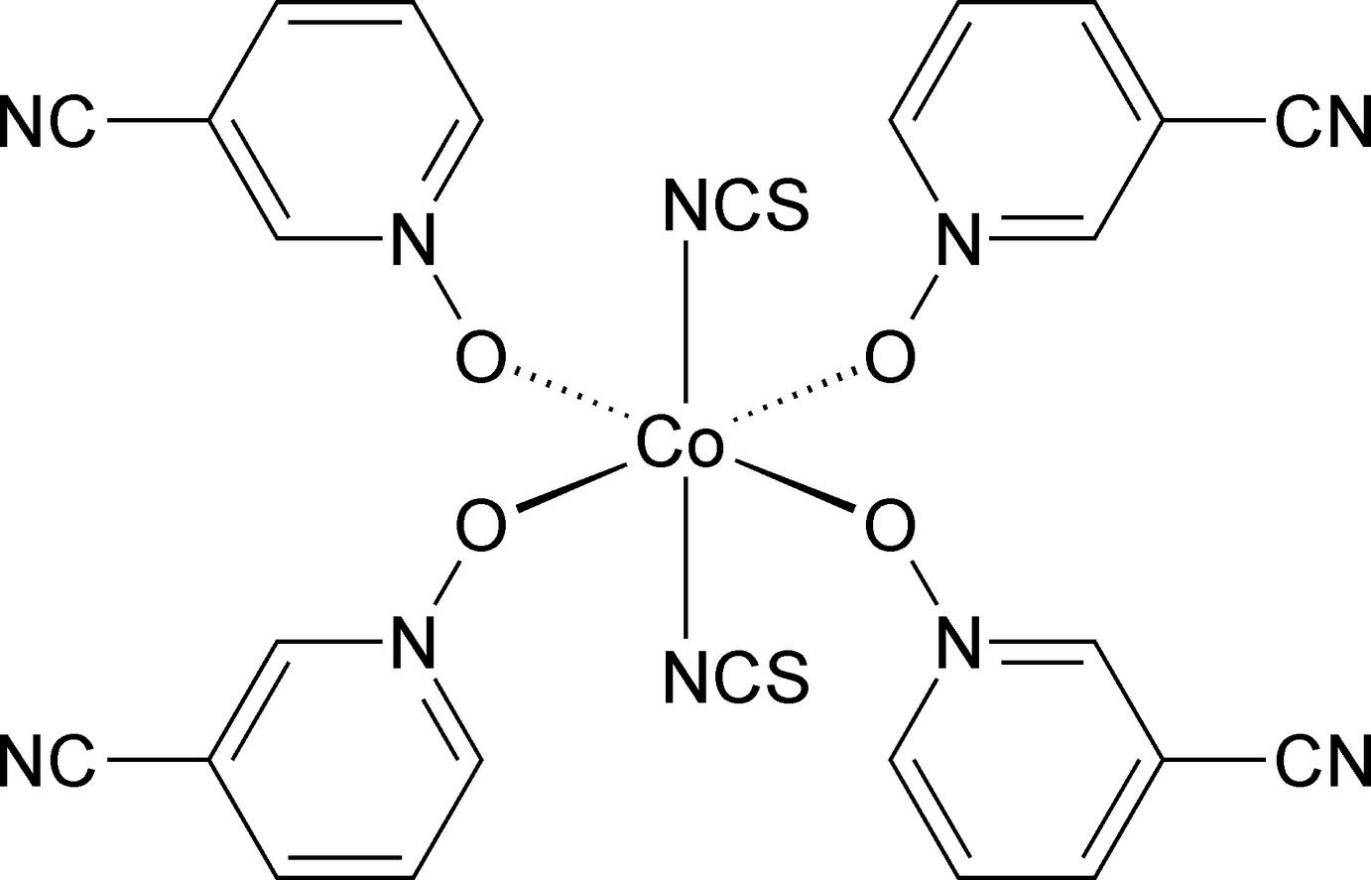


In recent investigations, N-donor coligands have been used that mostly consist of pyridine derivatives (Rams *et al.*, 2017 ▸), but to investigate the influence of the coligands on the magnetic anisotropy of Co^II^ centers, we also used S-donor coligands, such as ethyl­ene­thio­urea, that lead to a modified magnetic behavior (Jochim *et al.*, 2020 ▸). In a continuation of this work, we became inter­ested in O-donor coligands and we found that only very few Co(NCS)_2_ compounds with bridging thio­cyanate anions and such coligands have been reported in the literature (see *Database survey*). We also found that only in Co(NCS)_2_(THF)_2_ the Co^II^ cations are linked by pairs of bridging anionic ligands into linear chains (Cambridge Structural Database refcode QIKQUY; Shurdha *et al.*, 2013 ▸). In this context, we became inter­ested in pyridine *N*-oxide derivatives, for which two Co(NCS)_2_ compounds with bridging thio­cyanate anions are reported in the literature (see *Database survey*). In our first investigations, we used 3-cyano­pyridine *N*-oxide (C_6_H_4_N_2_O) as a coligand, which is commercially available and for which no coordination compounds have been reported. However, independent of the amounts of Co(NCS)_2_ and 3-cyano­pyridine *N*-oxide in the synthesis, the same crystalline phase was always obtained. The CN- stretching vibration of the cyano group is observed at 2241 cm^−1^ in the IR spectrum, indicating that this group is not involved in the metal coordination (Fig. S1). The CN-stretching vibration of the thio­cyanate anion occurs at 2051 cm^−1^, which proves that the anionic ligand is only terminally coordinated (Fig. S1). To confirm all these assumptions, the new crystalline phase was characterized by single crystal X-ray diffraction (see below).

## 2. Structural commentary

**Figure 1:**
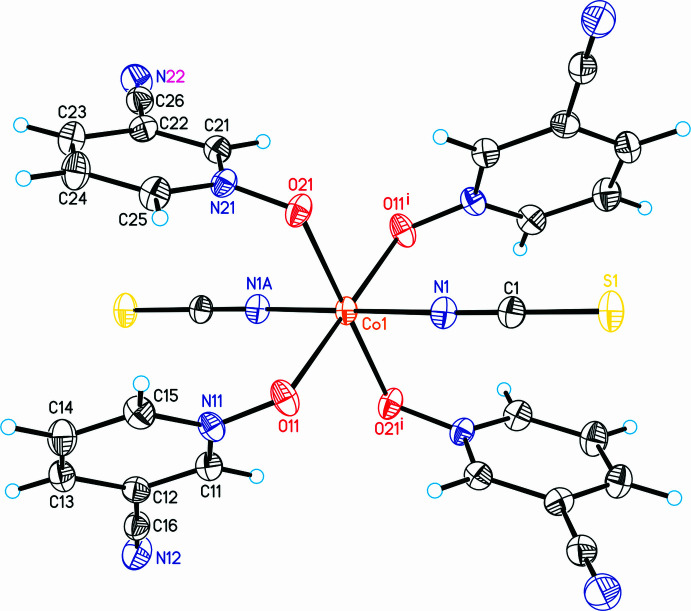
The mol­ecular structure of the title compound with labeling and displacement ellipsoids drawn at the 50% probability level. Symmetry code: (i) −*x*, −*y* + 1, −*z* + 1.

The asymmetric unit of the title compound, Co(SCN)_2_(C_6_H_4_N_2_O)_4_, consists of one crystallographically independent Co^II^ cation that is located on a center of inversion, as well as one independent thio­cyanate anion and two independent 3-cyano­pyridine *N*-oxide coligands in general positions (Fig. 1 ▸). The Co^II^ cations therefore adopt *trans*-CoN_2_O_4_ octa­hedral geometries (Fig. 1 ▸). Bond lengths and angles correspond to literature values and show that the octa­hedra are slightly distorted (Table 1 ▸).

**Figure 2:**
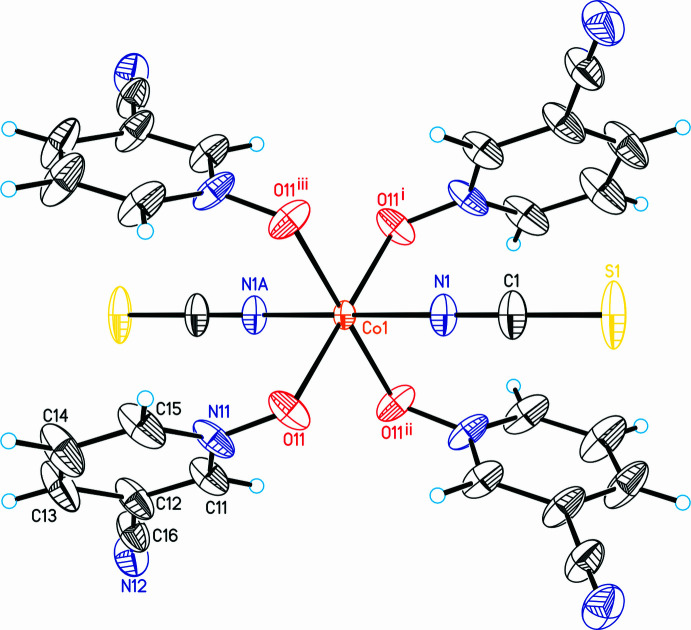
Mol­ecular structure of the title compound refined in space group *I*2/m with labeling and displacement ellipsoids drawn at the 50% probability level. Symmetry codes: (i) −*x*, 1 − *y*, 1 − *z*, (ii) −*x*, *y*, 1 − *z*, (iii) *x*, 1 − *y*, *z*.

If the structure is checked for higher symmetry using *PLATON* (Spek *et al.*, 2020 ▸) or *checkCIF*, a pseudo-translation and space group *I*2/*m* is suggested with 100% fit. The structure can easily be refined in this space group and the refinement leads to reasonable reliability factors. The refinement in space group *I*2/*m*, however, leads to significantly higher residuals than in space group *P*2_1_/*n* [*R*(*F*) for 2829 reflections with *F*_o_ > 4σ(*F*_o_) = 0.027 in *P*2_1_/*n versus* 0.033 for 1446 reflections with *F*_o_ > 4σ(*F*_o_) in *I*2/*m* and *wR*(*F* ^2^) = 0.083 for all 2829 independent reflections (*P*2_1_/*n*) versus 0.092 for all 1446 reflections (*I*2/*m*)]. In this context, it is noted that nearly all reflections violating the centering are observed. Moreover, from the refinement in *I*2/*m* it is obvious that significantly enlarged anisotropic displacement parameters are observed, which are much larger than expected for a measurement at 100 K, indicating too high symmetry (Fig. 2 ▸). For all these reasons, the crystal structure is presented in the monoclinic primitive space group *P*2_1_/*n*.

## 3. Supra­molecular features

**Figure 3:**
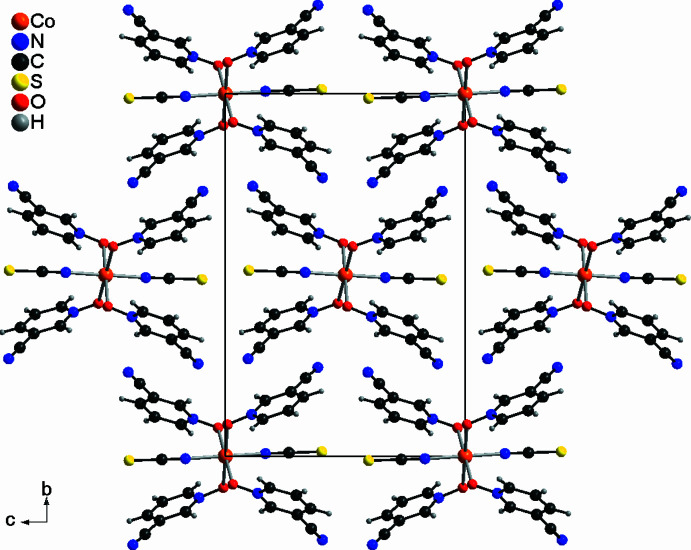
The packing of the title compound showing the arrangement of the discrete complexes along the crystallographic *a*-axis.

In the extended structure of the title compound, the complexes are arranged in columns that proceed along the crystallographic *a*-axis (Fig. 3 ▸). In this direction the translation leading to the pseudo-centering is also obvious. Several C—H⋯O, C—H⋯S and C—H⋯N contacts are observed between the complexes, but from the distances and angles it is obvious that they do not correspond to strong inter­actions (Table 2 ▸).

## 4. Thermoanalytical investigations

Based on the single-crystal data, an X-ray powder pattern was calculated and compared with the experimental pattern, which proves that the title compound was obtained as a pure phase (Fig. S2). Because in our synthetic investigations no further compounds were detected, it was checked whether a compound with a more condensed network is available by thermal ligand removal. Therefore, the title compound was investigated simultaneously by differential thermoanalysis and thermogravimetry under nitro­gen. Upon heating, only one mass loss of 58.8% is observed until 400°C that does not fit to a stepwise loss of the 3-cyano­pyridine *N*-oxide ligands (calculated mass loss for each 3-cyano­pyridine *N*-oxide ligand = 18.3%; Fig. S3). From the DTA curve, the onset of an endothermic event is visible, followed by a strong exothermic event at a peak temperature of 220°C. This is an unusual observation, because in previous investigations using pyridine derivatives the ligand removal always proceeds in an endothermic reaction. Oxidation of the compound might be excluded because all measurements were performed in a nitro­gen atmosphere and therefore one must assume that this ligand is thermally unstable and decomposes upon heating. In agreement with these observations, the residue obtained at 400°C is amorphous against X-rays (Fig. S4).

## 5. Database survey

A search in the Cambridge Structural Database (version 5.43, last update March 2023; Groom *et al.*, 2016 ▸) using ConQuest reveals that no coordination compounds with 3-cyano­pyridine *N*-oxide as a ligand have been reported. With 4-cyano­pyridine, three compounds are known, including 4-cyano­pyridine *N*-oxide)bis­(iso­thio­cyanate­aqua­zinc(II) (refcode UKEZIV; Mautner *et al.*, 2016 ▸) and the two isotypic compounds bis­(μ-thio­cyanato)-di­aqua-tetra­kis­(4-cyano­pyridine *N*-oxide)bis(iso­thio­cyanato) cadmium(II) (UKIMAE; Mautner *et al.*, 2016 ▸) and manganese(II) (KESSIN; Mautner *et al.*, 2018 ▸). The Zn compound consists of discrete complexes, in which the Zn^II^ cations are fivefold coordinated by two terminally N-bonded thio­cyanate anions, two 3-cyano­pyridine *N*-oxide ligands and one water mol­ecule, whereas the Mn and Cd compounds consist of dinuclear units, in which each metal cation is octa­hedrally coordinated by one water mol­ecule, one terminal and two bridging thio­cyanate anions and two 4-cyano­pyridine *N*-oxide ligands, and are linked into dinuclear units by pairs of μ-1,3-bridging thio­cyanate anions.

Some compounds based on Co(NCS)_2_ and pyridine *N*-oxide derivatives in which the Co^II^ cations are linked by μ-1,3-bridging thio­cyanate anions are also known. This include the two isotypic compounds (4-methyl­pyridine *N*-oxide)bis­thio­cyanate)­cobalt(II) (MEQKOJ; Zhang *et al.*, 2006*b* ▸) (4-meth­oxy­pyridine *N*-oxide)bis­thio­cyanate)­cobalt(II) (TERRAK; Zhang *et al.*, 2006*a* ▸), (4-methyl­pyridine *N*-oxide)(meth­anol)bis­thio­cyanate)­cobalt(II) (REKBUF; Shi *et al.*, 2006 ▸) and bis­(4-nitro­pyridine *N*-oxide)bis­(thio­cyanate)­cobalt(II) (TILHIG; Shi *et al.*, 2007 ▸). In the first two compounds, the Co^II^ cations are linked by pairs of thio­cyanate anions into corrugated chains that are further connected into layers by μ-1,1(*O*,*O*) bridging coligands. In the third compound with methanol, two Co^II^ cations are linked by pairs of anionic ligands into dinuclear units and are further linked by pairs of μ-1,1(*O*,*O*) bridging 4-nitro­pyridine *N*-oxide ligands. In the compound with the 4-nitro substituent, the cations are linked by pairs of bridging thio­cyanate anions into chains that are corrugated because of the *cis*–*cis*–*trans* configuration at the Co^II^ centers.

## 6. Synthesis and crystallization

Co(NCS)_2_ (99%) was purchased from Sigma Aldrich, 3-cyano­pyridine *N*-oxide (97%) was purchased from Thermo Scientific and ethanol (99.9%) was purchased from Fisher Chemical.

**Synthesis:**

Single crystals were obtained by the reaction of 0.25 mmol (43.5 mg) Co(SCN)_2_ and 1 mmol (120 mg) 3-cyano­pyridine *N*-oxide in 1 ml of ethanol. The reaction mixture was stored overnight, which lead to the formation of yellow needle-like crystals.

For the preparation of larger amounts of a microcrystalline powder, the same amount of reactants were stirred in 2 ml of ethanol for 1 d.

**Experimental details:**

The PXRD measurements were performed with a Stoe Transmission Powder Diffraction System (STADI P) equipped with a MYTHEN 1K detector and a Johansson-type Ge(111) monochromator using Cu *K*α_1_ radiation (λ = 1.540598 Å).

The IR spectra were measured using an ATI Mattson Genesis Series FTIR Spectrometer, control software: WINFIRST, from ATI Mattson.

Thermogravimetry and differential thermoanalysis (TG–DTA) measurements were performed in a dynamic nitro­gen atmosphere in Al_2_O_3_ crucibles using a STA-PT 1000 thermobalance from Linseis. The instrument was calibrated using standard reference materials.

## 7. Refinement

Crystal data, data collection and structure refinement details are summarized in Table 3 ▸. The hydrogen atoms were positioned with idealized geometry and were refined with *U*_iso_(H) = 1.2*U*_eq_(C) using a riding model. As mentioned in the *Structural commentary*, the compound shows strong pseudosymmetry because of a pseudo-lattice translation indicating a centering, but our investigations show that the structure is best described in the primitive space group *P*2_1_/*n* instead of *I*2/*m*. This is obvious in the reliability factors obtained by refinements in both space groups, but especially from the large components of the anisotropic displacement parameters if the structure is refined in the body-centered space group. Moreover, nearly all of the reflections that would violate the centering were observed.

## Supplementary Material

Crystal structure: contains datablock(s) I. DOI: 10.1107/S2056989023006862/hb8070sup1.cif

Structure factors: contains datablock(s) I. DOI: 10.1107/S2056989023006862/hb8070Isup2.hkl

Fig. S1. IR spectrum of the title compound. Given is the value of the CN stretching vibration of the cyanao group and the thiocyanate anion. DOI: 10.1107/S2056989023006862/hb8070sup3.png

Fig. S2. Experimental (top) and calculated (bottom) X-ray powder pattern of the title compound. DOI: 10.1107/S2056989023006862/hb8070sup4.png

Fig. S3. DTG (top), TG (middle) and DTA curve (bottom) of the title compound measured with 4C/min. DOI: 10.1107/S2056989023006862/hb8070sup5.png

Fig. S4. Experimental X-ray powder pattern of the residue obtained after the first mass loss, in a thermogravimetric measurement of the title compound. DOI: 10.1107/S2056989023006862/hb8070sup6.png

CCDC reference: 2286746

Additional supporting information:  crystallographic information; 3D view; checkCIF report

## Figures and Tables

**Table 1:** Selected geometric parameters (Å, °)

Co1—N1	2.0596 (13)	Co1—O21	2.0985 (9)
Co1—O11	2.1019 (10)		
			
N1^i^—Co1—O11	93.52 (4)	N1—Co1—O21^i^	94.51 (4)
N1—Co1—O11	86.48 (4)	O21—Co1—O11	90.62 (4)
N1—Co1—O21	85.49 (4)	O21—Co1—O11^i^	89.38 (4)

Symmetry code: (i) 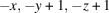 .

**Table 2:** Hydrogen-bond geometry (Å, °)

*D*—H⋯*A*	*D*—H	H⋯*A*	*D*⋯*A*	*D*—H⋯*A*
C11—H11⋯O21^i^	0.95	2.61	3.3295 (17)	133
C11—H11⋯N22^ii^	0.95	2.57	3.3902 (19)	145
C14—H14⋯S1^iii^	0.95	2.91	3.7585 (15)	149
C15—H15⋯O21^iii^	0.95	2.45	3.2351 (16)	140
C21—H21⋯N12^iv^	0.95	2.52	3.3233 (19)	142
C24—H24⋯S1^iii^	0.95	2.96	3.8082 (15)	150
C25—H25⋯O11^iii^	0.95	2.28	3.1335 (16)	148

Symmetry codes: (i) 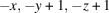 ; (ii) 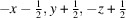 ; (iii) 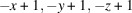 ; (iv) 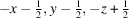 .

**Table 3:** Experimental details

Crystal data	
Chemical formula	[Co(NCS)_2_(C_6_H_4_N_2_O)_4_]
*M*_r_	655.54
Crystal system, space group	Monoclinic, *P*2_1_/*n*
Temperature (K)	100
*a*, *b*, *c* (Å)	6.5899 (1), 17.9658 (2), 11.9444 (1)
β (°)	96.131 (1)
*V* (Å^3^)	1406.04 (3)
*Z*	2
Radiation type	Cu *K*α
μ (mm^−1^)	6.63
Crystal size (mm)	0.28 × 0.03 × 0.03

Data collection	
Diffractometer	XtaLAB Synergy, Dualflex, HyPix
Absorption correction	Multi-scan (*CrysAlis PRO*; Rigaku OD, 2022 ▸)
*T*_min_, *T*_max_	0.571, 1.000
No. of measured, independent and observed [*I* > 2σ(*I*)] reflections	23719, 2990, 2829
*R*_int_	0.028
(sin θ/λ)_max_ (Å^−1^)	0.635

Refinement	
*R*[*F* ^2^ > 2σ(*F* ^2^)], *wR*(*F* ^2^), *S*	0.027, 0.083, 1.11
No. of reflections	2990
No. of parameters	197
H-atom treatment	H-atom parameters constrained
Δρ_max_, Δρ_min_ (e Å^−3^)	0.24, −0.34

Computer programs: *CrysAlis PRO* (Rigaku OD, 2022 ▸), *SHELXT2014/5* (Sheldrick, 2015*b* ▸), *SHELXL2016/6* (Sheldrick, 2015*a* ▸), *DIAMOND* (Brandenburg & Putz, 1999 ▸) and *publCIF* (Westrip, 2010 ▸).

## supplementary crystallographic information

### Crystal data

**Table:**

[Co(NCS)_2_(C_6_H_4_N_2_O)_4_]	*F*(000) = 666
*M**_r_* = 655.54	*D*_x_ = 1.548 Mg m^−^^3^
Monoclinic, *P*2_1_/*n*	Cu *K*α radiation, λ = 1.54184 Å
*a* = 6.5899 (1) Å	Cell parameters from 16616 reflections
*b* = 17.9658 (2) Å	θ = 4.5–76.3°
*c* = 11.9444 (1) Å	µ = 6.63 mm^−^^1^
β = 96.131 (1)°	*T* = 100 K
*V* = 1406.04 (3) Å^3^	Needle, yellow
*Z* = 2	0.28 × 0.03 × 0.03 mm

### Data collection

**Table:**

XtaLAB Synergy, Dualflex, HyPix diffractometer	2990 independent reflections
Radiation source: micro-focus sealed X-ray tube, PhotonJet (Cu) X-ray Source	2829 reflections with *I* > 2σ(*I*)
Mirror monochromator	*R*_int_ = 0.028
Detector resolution: 10.0000 pixels mm^-1^	θ_max_ = 78.3°, θ_min_ = 4.5°
ω scans	*h* = −8→7
Absorption correction: multi-scan (CrysAlisPro; Rigaku OD, 2022)	*k* = −22→22
*T*_min_ = 0.571, *T*_max_ = 1.000	*l* = −13→15
23719 measured reflections	

### Refinement

**Table:**

Refinement on *F*^2^	Hydrogen site location: inferred from neighbouring sites
Least-squares matrix: full	H-atom parameters constrained
*R*[*F*^2^ > 2σ(*F*^2^)] = 0.027	*w* = 1/[σ^2^(*F*_o_^2^) + (0.0511*P*)^2^ + 0.3714*P*] where *P* = (*F*_o_^2^ + 2*F*_c_^2^)/3
*wR*(*F*^2^) = 0.083	(Δ/σ)_max_ = 0.001
*S* = 1.11	Δρ_max_ = 0.24 e Å^−^^3^
2990 reflections	Δρ_min_ = −0.34 e Å^−^^3^
197 parameters	Extinction correction: *SHELXL2016/6* (Sheldrick, 2015a), Fc^*^=kFc[1+0.001xFc^2^λ^3^/sin(2θ)]^-1/4^
0 restraints	Extinction coefficient: 0.0007 (2)
Primary atom site location: dual	

### Special details

**Table:**

Geometry. All esds (except the esd in the dihedral angle between two l.s. planes) are estimated using the full covariance matrix. The cell esds are taken into account individually in the estimation of esds in distances, angles and torsion angles; correlations between esds in cell parameters are only used when they are defined by crystal symmetry. An approximate (isotropic) treatment of cell esds is used for estimating esds involving l.s. planes.

### Fractional atomic coordinates and isotropic or equivalent isotropic displacement parameters (Å^2^)

**Table:**

	*x*	*y*	*z*	*U*_iso_*/*U*_eq_	
Co1	0.000000	0.500000	0.500000	0.01595 (11)	
N1	0.1137 (2)	0.50708 (6)	0.66710 (11)	0.0204 (3)	
C1	0.1405 (2)	0.50936 (7)	0.76538 (13)	0.0196 (3)	
S1	0.17656 (6)	0.51232 (2)	0.90206 (3)	0.02903 (12)	
O11	0.22429 (14)	0.57834 (6)	0.46915 (8)	0.0237 (2)	
N11	0.23654 (17)	0.60259 (6)	0.36497 (9)	0.0198 (2)	
C11	0.0786 (2)	0.64024 (8)	0.31076 (11)	0.0208 (3)	
H11	−0.037683	0.652022	0.348001	0.025*	
C12	0.0881 (2)	0.66154 (8)	0.19988 (11)	0.0214 (3)	
C13	0.2587 (2)	0.64494 (8)	0.14458 (12)	0.0246 (3)	
H13	0.264095	0.658366	0.068026	0.030*	
C14	0.4198 (2)	0.60828 (9)	0.20484 (13)	0.0270 (3)	
H14	0.539395	0.597242	0.170098	0.032*	
C15	0.4073 (2)	0.58770 (8)	0.31525 (12)	0.0242 (3)	
H15	0.518894	0.563014	0.356478	0.029*	
C16	−0.0832 (2)	0.70028 (8)	0.14185 (12)	0.0246 (3)	
N12	−0.2185 (2)	0.73137 (8)	0.09481 (11)	0.0324 (3)	
O21	0.20988 (14)	0.41291 (6)	0.49113 (8)	0.0221 (2)	
C21	0.0785 (2)	0.34632 (7)	0.33413 (12)	0.0206 (3)	
H21	−0.041400	0.335659	0.368791	0.025*	
C22	0.0971 (2)	0.32231 (8)	0.22576 (12)	0.0218 (3)	
C23	0.2739 (2)	0.33654 (8)	0.17478 (12)	0.0263 (3)	
H23	0.286155	0.320817	0.099860	0.032*	
C24	0.4310 (2)	0.37428 (9)	0.23672 (13)	0.0279 (3)	
H24	0.554515	0.383844	0.204898	0.034*	
C25	0.4087 (2)	0.39810 (8)	0.34478 (12)	0.0239 (3)	
H25	0.517252	0.423673	0.387264	0.029*	
C26	−0.0726 (2)	0.28445 (8)	0.16431 (12)	0.0247 (3)	
N22	−0.2080 (2)	0.25501 (8)	0.11394 (12)	0.0323 (3)	
N21	0.23268 (17)	0.38504 (6)	0.39006 (9)	0.0190 (2)	

### Atomic displacement parameters (Å^2^)

**Table:**

	*U*^11^	*U*^22^	*U*^33^	*U*^12^	*U*^13^	*U*^23^
Co1	0.01140 (17)	0.02239 (17)	0.01407 (18)	0.00014 (9)	0.00137 (12)	−0.00016 (10)
N1	0.0170 (6)	0.0261 (6)	0.0178 (6)	0.0005 (4)	0.0010 (5)	−0.0008 (4)
C1	0.0135 (6)	0.0248 (6)	0.0207 (7)	−0.0004 (4)	0.0032 (5)	−0.0009 (5)
S1	0.0330 (2)	0.0394 (2)	0.01490 (19)	−0.00001 (15)	0.00368 (14)	−0.00104 (13)
O11	0.0188 (4)	0.0343 (5)	0.0174 (5)	−0.0063 (4)	−0.0012 (3)	0.0061 (4)
N11	0.0162 (5)	0.0236 (5)	0.0193 (5)	−0.0040 (4)	0.0009 (4)	0.0026 (4)
C11	0.0167 (6)	0.0240 (6)	0.0216 (6)	−0.0005 (5)	0.0014 (5)	0.0002 (5)
C12	0.0208 (6)	0.0205 (6)	0.0230 (7)	−0.0007 (5)	0.0016 (5)	0.0004 (5)
C13	0.0256 (7)	0.0284 (7)	0.0203 (6)	−0.0024 (5)	0.0044 (5)	0.0011 (5)
C14	0.0192 (6)	0.0348 (8)	0.0279 (7)	0.0011 (5)	0.0057 (5)	0.0002 (6)
C15	0.0172 (6)	0.0279 (7)	0.0275 (7)	−0.0008 (5)	0.0018 (5)	0.0021 (5)
C16	0.0276 (7)	0.0264 (7)	0.0202 (6)	0.0020 (5)	0.0038 (5)	0.0001 (5)
N12	0.0348 (7)	0.0369 (7)	0.0250 (6)	0.0122 (6)	0.0009 (5)	0.0006 (5)
O21	0.0200 (5)	0.0295 (5)	0.0165 (4)	0.0058 (4)	0.0000 (3)	−0.0042 (4)
C21	0.0165 (6)	0.0222 (6)	0.0230 (6)	−0.0001 (5)	0.0022 (5)	0.0011 (5)
C22	0.0214 (6)	0.0209 (6)	0.0227 (6)	0.0009 (5)	0.0003 (5)	−0.0010 (5)
C23	0.0239 (7)	0.0324 (7)	0.0232 (7)	0.0005 (6)	0.0053 (5)	−0.0035 (6)
C24	0.0197 (6)	0.0380 (8)	0.0268 (7)	−0.0013 (6)	0.0057 (5)	−0.0031 (6)
C25	0.0158 (6)	0.0294 (7)	0.0261 (7)	−0.0005 (5)	0.0010 (5)	−0.0022 (5)
C26	0.0273 (7)	0.0244 (7)	0.0226 (7)	−0.0020 (5)	0.0030 (5)	−0.0002 (5)
N22	0.0343 (7)	0.0333 (7)	0.0286 (6)	−0.0102 (6)	0.0001 (5)	−0.0022 (5)
N21	0.0168 (5)	0.0219 (5)	0.0181 (5)	0.0034 (4)	0.0010 (4)	−0.0020 (4)

### Geometric parameters (Å, º)

**Table:**

Co1—N1^i^	2.0596 (13)	C14—H14	0.9500
Co1—N1	2.0596 (13)	C14—C15	1.381 (2)
Co1—O11	2.1019 (10)	C15—H15	0.9500
Co1—O11^i^	2.1019 (10)	C16—N12	1.147 (2)
Co1—O21^i^	2.0985 (9)	O21—N21	1.3302 (14)
Co1—O21	2.0985 (9)	C21—H21	0.9500
N1—C1	1.169 (2)	C21—C22	1.382 (2)
C1—S1	1.6254 (16)	C21—N21	1.3480 (17)
O11—N11	1.3290 (14)	C22—C23	1.395 (2)
N11—C11	1.3478 (17)	C22—C26	1.4410 (19)
N11—C15	1.3540 (18)	C23—H23	0.9500
C11—H11	0.9500	C23—C24	1.384 (2)
C11—C12	1.3864 (18)	C24—H24	0.9500
C12—C13	1.3956 (19)	C24—C25	1.382 (2)
C12—C16	1.4398 (19)	C25—H25	0.9500
C13—H13	0.9500	C25—N21	1.3519 (18)
C13—C14	1.384 (2)	C26—N22	1.150 (2)
			
N1^i^—Co1—N1	180.0	C14—C13—H13	121.1
N1^i^—Co1—O11^i^	86.48 (4)	C13—C14—H14	119.9
N1^i^—Co1—O11	93.52 (4)	C15—C14—C13	120.24 (13)
N1—Co1—O11	86.48 (4)	C15—C14—H14	119.9
N1—Co1—O11^i^	93.52 (4)	N11—C15—C14	120.21 (13)
N1^i^—Co1—O21^i^	85.49 (4)	N11—C15—H15	119.9
N1—Co1—O21	85.49 (4)	C14—C15—H15	119.9
N1^i^—Co1—O21	94.51 (4)	N12—C16—C12	179.29 (17)
N1—Co1—O21^i^	94.51 (4)	N21—O21—Co1	117.74 (7)
O11^i^—Co1—O11	180.00 (3)	C22—C21—H21	120.4
O21—Co1—O11	90.62 (4)	N21—C21—H21	120.4
O21—Co1—O11^i^	89.38 (4)	N21—C21—C22	119.20 (12)
O21^i^—Co1—O11^i^	90.62 (4)	C21—C22—C23	120.87 (13)
O21^i^—Co1—O11	89.38 (4)	C21—C22—C26	118.88 (13)
O21^i^—Co1—O21	180.0	C23—C22—C26	120.21 (13)
C1—N1—Co1	167.31 (12)	C22—C23—H23	121.0
N1—C1—S1	179.70 (15)	C24—C23—C22	117.94 (13)
N11—O11—Co1	119.86 (7)	C24—C23—H23	121.0
O11—N11—C11	119.56 (11)	C23—C24—H24	119.9
O11—N11—C15	118.84 (11)	C25—C24—C23	120.19 (13)
C11—N11—C15	121.60 (12)	C25—C24—H24	119.9
N11—C11—H11	120.5	C24—C25—H25	120.0
N11—C11—C12	119.07 (12)	N21—C25—C24	120.07 (13)
C12—C11—H11	120.5	N21—C25—H25	120.0
C11—C12—C13	120.94 (13)	N22—C26—C22	178.95 (16)
C11—C12—C16	118.69 (12)	O21—N21—C21	119.46 (11)
C13—C12—C16	120.36 (12)	O21—N21—C25	118.86 (11)
C12—C13—H13	121.1	C21—N21—C25	121.66 (12)
C14—C13—C12	117.87 (13)		

Symmetry code: (i) −*x*, −*y*+1, −*z*+1.

### Hydrogen-bond geometry (Å, º)

**Table:**

*D*—H···*A*	*D*—H	H···*A*	*D*···*A*	*D*—H···*A*
C11—H11···O21^i^	0.95	2.61	3.3295 (17)	133
C11—H11···N22^ii^	0.95	2.57	3.3902 (19)	145
C14—H14···S1^iii^	0.95	2.91	3.7585 (15)	149
C15—H15···O21^iii^	0.95	2.45	3.2351 (16)	140
C21—H21···N12^iv^	0.95	2.52	3.3233 (19)	142
C24—H24···S1^iii^	0.95	2.96	3.8082 (15)	150
C25—H25···O11^iii^	0.95	2.28	3.1335 (16)	148

Symmetry codes: (i) −*x*, −*y*+1, −*z*+1; (ii) −*x*−1/2, *y*+1/2, −*z*+1/2; (iii) −*x*+1, −*y*+1, −*z*+1; (iv) −*x*−1/2, *y*−1/2, −*z*+1/2.
