# Supplementary figures and images for: Synthesis, crystal structure and thermal behavior of tetra­kis­(3-cyano­pyridine N-oxide-κO)bis­(thio­cyanato-κN)cobalt(II), which shows strong pseudo­symmetry
Source: Acta Crystallogr E Crystallogr Commun. 2023 Sep 8;79(Pt 10):867–71. doi: 10.1107/S2056989023006862 (PMC10561199; doi:10.1107/S2056989023006862)

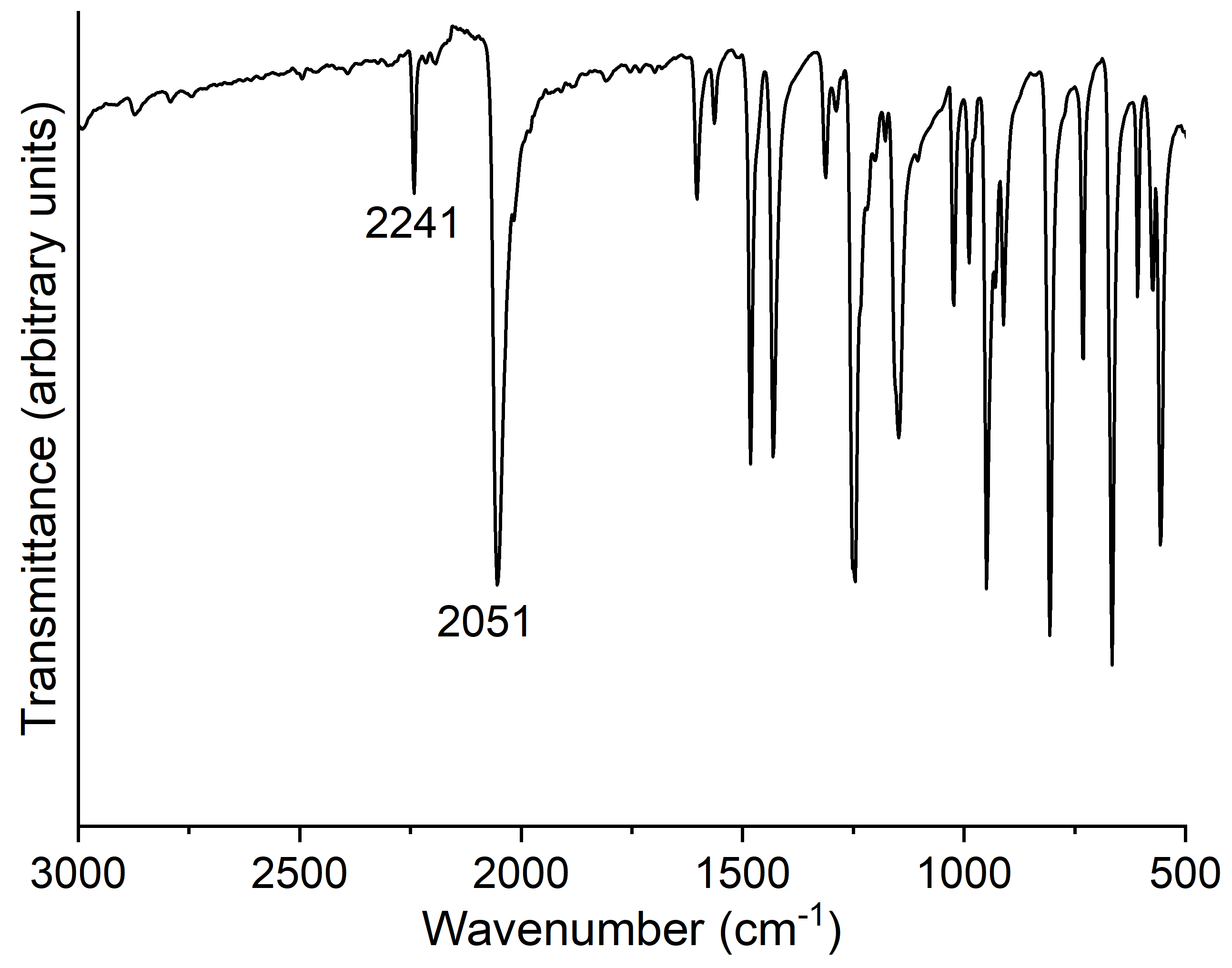

Supplement: Supplementary file 3 [file e-79-00867-sup3.png]

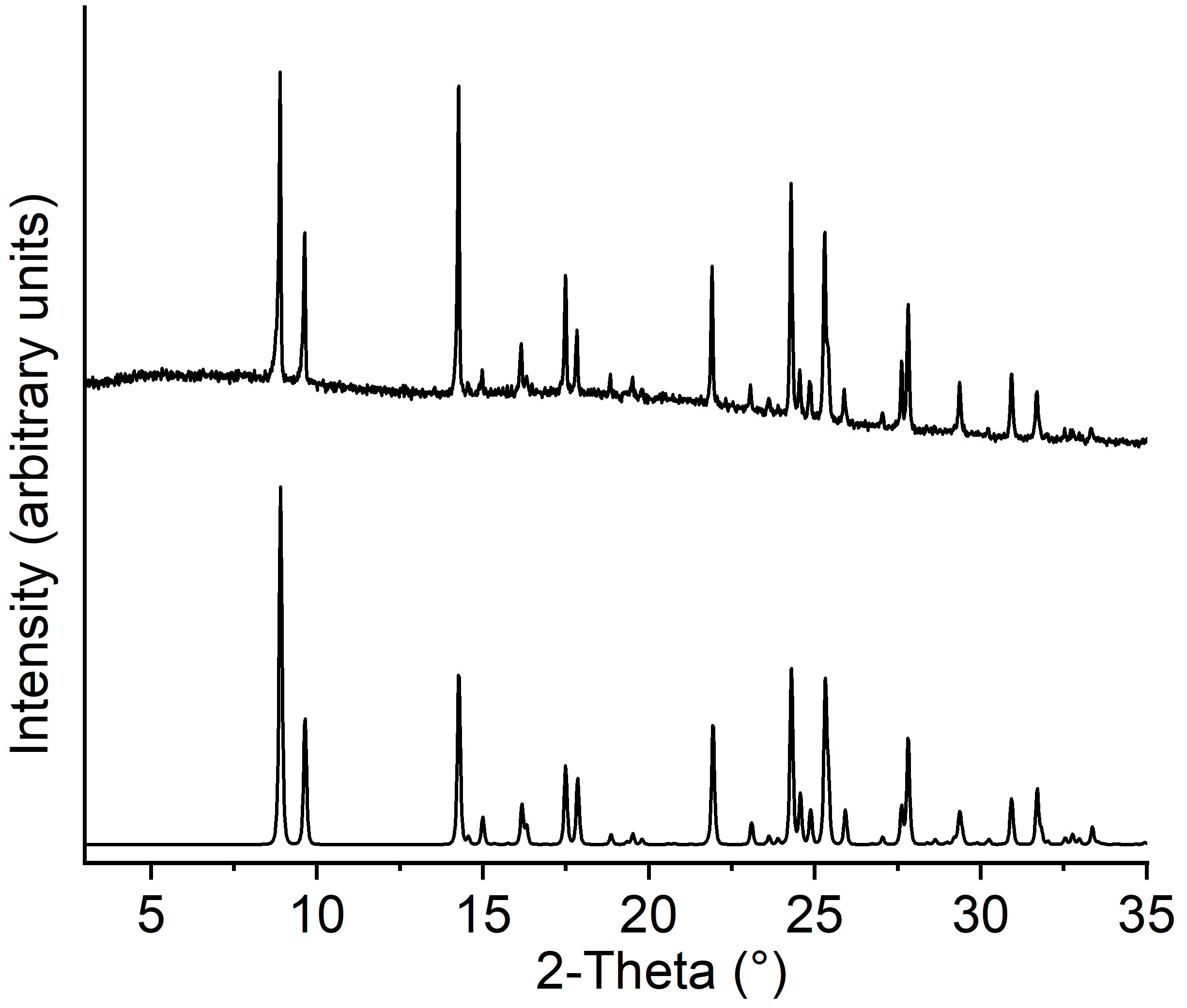

Supplement: Supplementary file 4 [file e-79-00867-sup4.png]

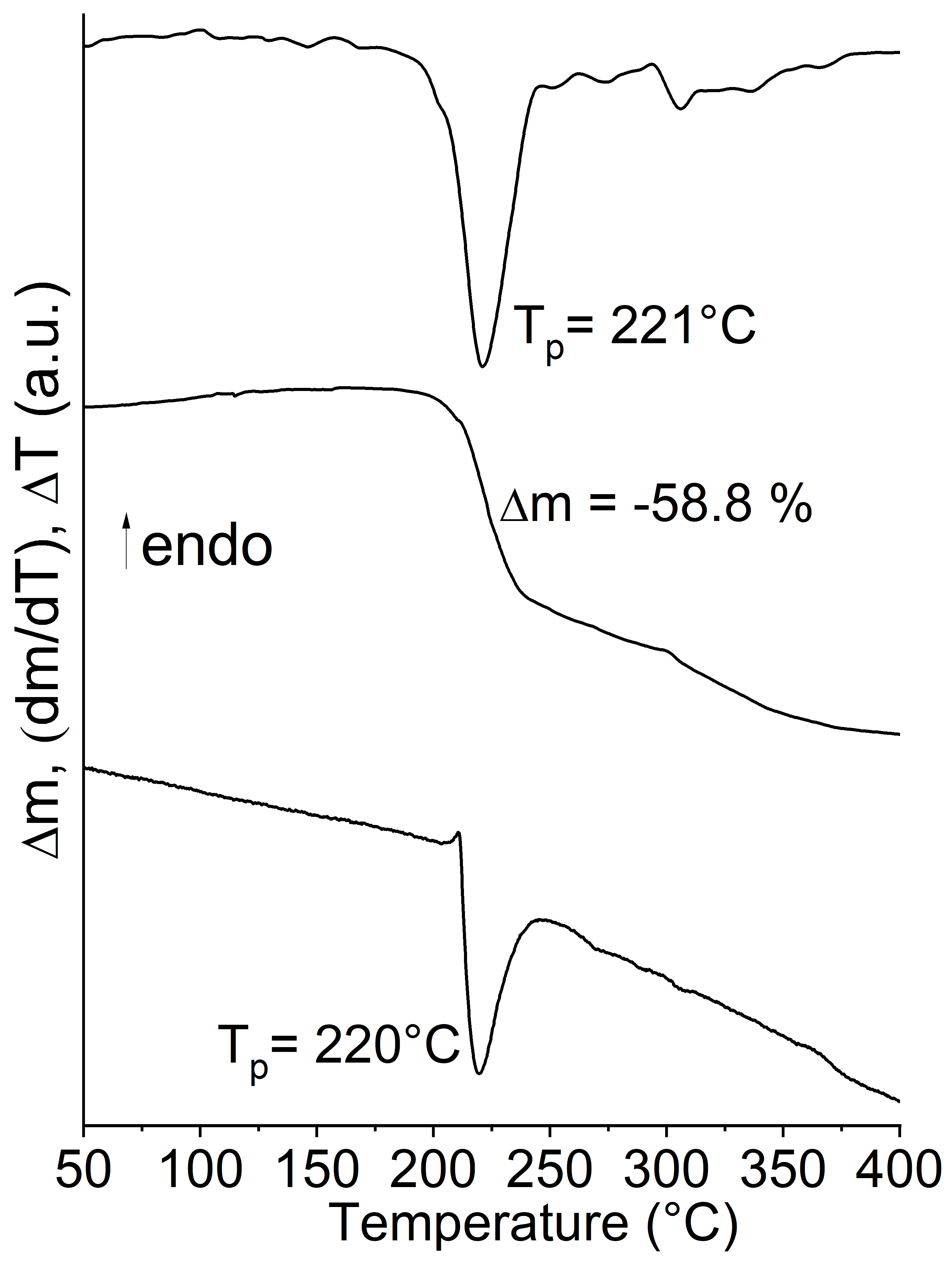

Supplement: Supplementary file 5 [file e-79-00867-sup5.png]

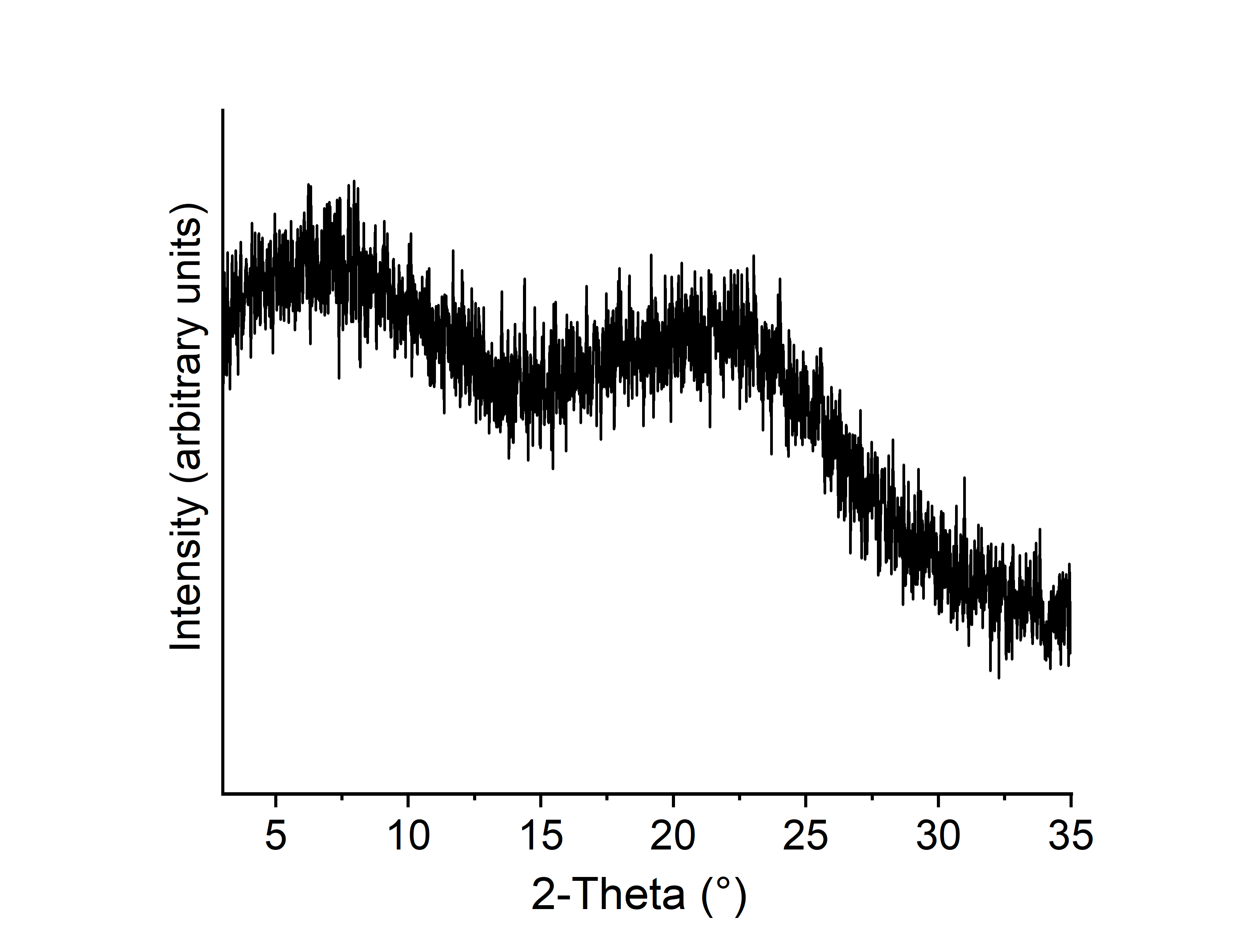

Supplement: Supplementary file 6 [file e-79-00867-sup6.png]
